# Unacceptable failure of osteochondral glenoid allograft for biologic resurfacing of the glenoid

**DOI:** 10.1186/s40634-021-00419-x

**Published:** 2021-12-02

**Authors:** Filippo Familiari, Bettina Hochreiter, Christian Gerber

**Affiliations:** 1 Department of Orthopaedics, Balgrist University Hospital, University of Zurich, Zürich, Switzerland; 2 Department of Orthopaedic and Trauma Surgery, Magna Graecia University, Catanzaro, Italy

**Keywords:** Shoulder osteoarthritis, young patients, osteochondral glenoid allograft, biologic resurfacing, failure rate, Constant and Murley score

## Abstract

**Purpose:**

Glenohumeral osteoarthritis (OA) represents a challenging problem in young, physically active patients. It was the purpose of this investigation to evaluate the results of a pilot study involving glenoid resurfacing with a glenoid allograft combined with a hemiarthroplasty on the humeral side.

**Methods:**

Between April 2011 to November 2013, 5 patients (3 men, 2 women, mean age 46.4, range 35-57) with advanced OA of the glenohumeral joint, were treated with a humeral head replacement combined with replacement of the glenoid surface with an osteochondral, glenoid allograft.

**Results:**

Overall, clinically, there was one excellent, one satisfactory and three poor results. Mean preoperative subjective shoulder value (SSV) was 34% (range: 20-50%) and preoperative relative Constant-Murley-Score (CSr) was 43 points (range: 29-64 points). Three patients with poor results had to be revised within the first three years. Their mean pre-revision SSV and CSr were 38% (range: 15-80%) and 36 points (range: 7-59 points) respectively. One patient was revised 9 years after the primary procedure with advanced glenoid erosion and pain and one patient has an ongoing satisfactory outcome without revision. Their SSVs were 60% and 83%, their CSr were 65 points and 91 points, 9 and 10 years after the primary procedure, respectively. Mean follow-up was 7 years (2-10 years) and mean time to revision was 4 years (range: 1-9 years).

**Conclusion:**

The in-vivo pilot study of a previously established in-vitro technique of osteochondral glenoid allograft combined with humeral HA led to three early failures and only one really satisfactory clinical outcome which, however, was associated with advanced glenoid erosion. Osteochondral allograft glenoid resurfacing was associated with an unacceptable early failure rate and no results superior to those widely documented for HA or TSA, so that the procedure has been abandoned.

**Level of evidence:**

Level IV, Case Series, Treatment Study.

## Introduction

The management of the young and active patient with primary, posttraumatic or postoperative glenohumeral osteoarthritis (OA) remains controversial. For advanced cases, hemi- (HA) and total shoulder arthroplasty (TSA) are the preferred solutions with better active mobility, pain relief, and patient satisfaction with TSA than with HA [1, 2]. Both surgical solutions, however, are not entirely satisfactory: for TSA loosening of the glenoid component remains a too frequent cause of failure associated with revision in 0.8% of the cases per year [3–5]; and HA may be associated with progressive glenoid erosion and unsatisfactory clinical outcome in the young individual [6, 7].

Multiple interpositional soft-tissue grafts have been proposed to avoid or defer glenoid replacement including fascia lata, Achilles tendon allograft, anterior capsule, lateral meniscal allograft, and several dermal allografts [8–11]. Originally introduced by Burkhead and Hutton in 1995 [12], interpositional soft tissue glenoid arthroplasty, combined with HA, has been proposed for glenohumeral OA in young patients. Subsequently, conflicting results have been reported [10, 13]. Presently, the true value of biologic resurfacing to preserve glenoid bone stock, specifically its comparison with modern TSA, remains undetermined [11, 14].

The concept of articular allograft replacement is well established in different fields of orthopedic surgery [15–18], with good mid- to long-term results especially in unipolar joint disease [19, 20]. To assess the potential of glenoid allograft replacement associated with arthritic humeral head resurfacing, nine potential geometric designs of a scapular neck-glenoid allograft constructs were experimentally tested [21]. A “rectangle” design was determined to be best and was therefore chosen for five clinical pilot cases. The purpose of this clinical pilot study was to investigate the results of glenoid allograft resurfacing combined with a prosthetic humeral head replacement on the basis of positive in-vitro results and because the currently preferred options HA and TSA are not yet considered a safe and durable solution.

## Materials and methods

Institutional review board approval was obtained for this retrospective study (BASEC-Nr. Req-2017-00702).

### Study group

From April 2011 to November 2013, 5 patients underwent a HA with an osteochondral glenoid allograft for the treatment of glenohumeral OA. The 5 patients (3 men and 2 women) were of a mean age of 46.4 (range, 35-57) years at surgery. The dominant arm was involved in 2 of the 5 patients.

### Surgical technique

**Fig. 1:**
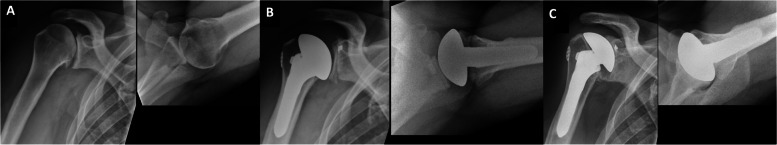
Radiological FU imaging of the patient with a satisfactory course. Preoperatively (**A**), 1 year postoperatively (**B**), and 10 years postoperatively (**C**)

**Fig. 2:**
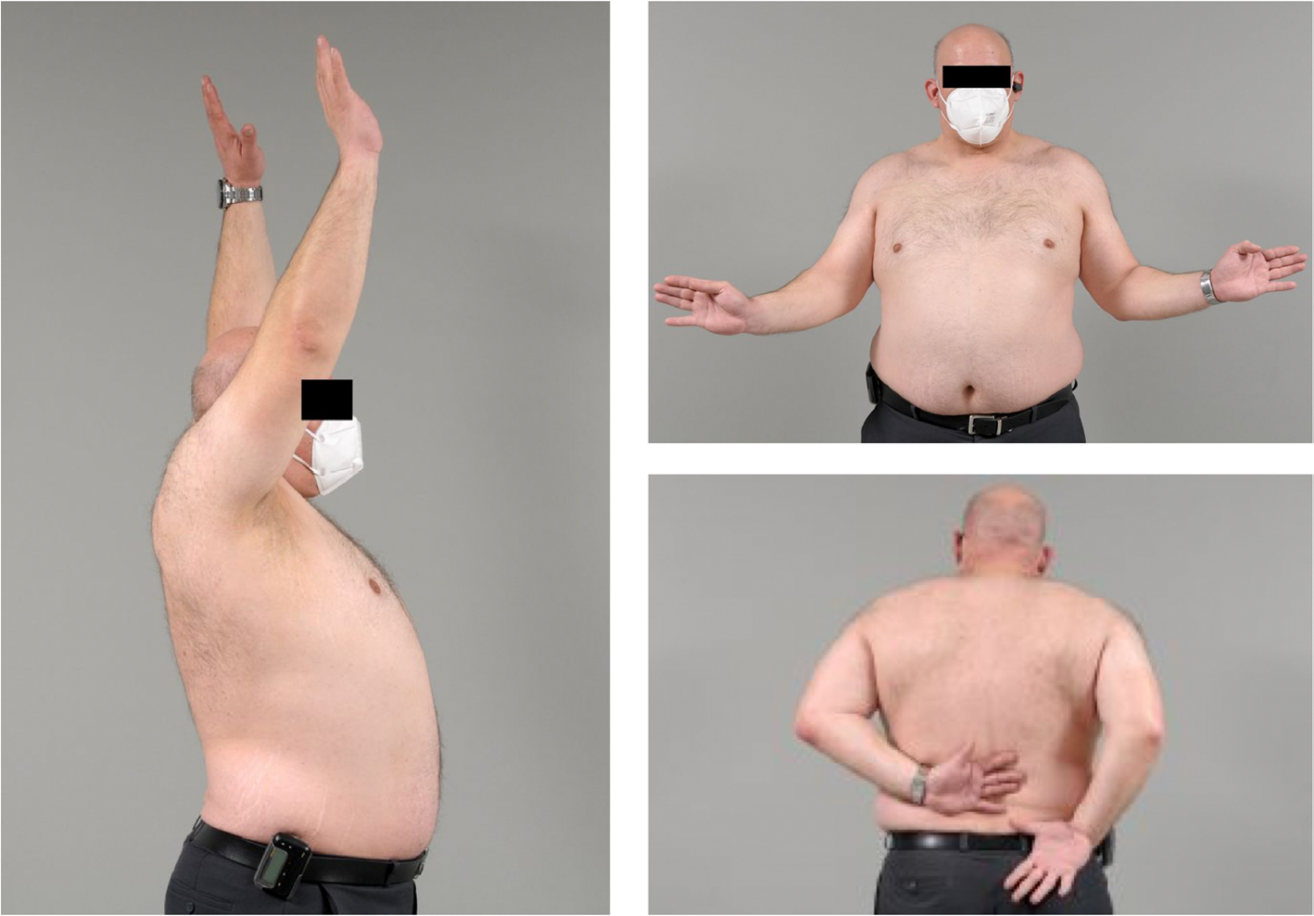
Clinical imaging of the patient with a satisfactory course at 10-year FU with advanced glenoid erosion but almost unlimited clinical function

All patients received perioperative antibiotic prophylaxis. All surgeries were performed in a beach chair position under interscalene block by the same surgeon (CG). Through a deltopectoral approach the joint was approached using a lesser tuberosity osteotomy [22]. The subscapularis was mobilised, and the humeral head was exposed. A biceps tenotomy was performed routinely. The arthritic humeral head was resected anatomically after removing osteophytes. The glenoid was exposed with an anterior and posterior capsular release and the labrum was circumferentially excised. The glenoid surface was reamed flat. The size- and side matched, non arthritic, fresh-frozen glenoid allograft which had been procured from an accredited tissue bank, was prepared using a custom made cutting block. It was then slowly thawed in saline solution of body temperature. The glenoid peg hole was prepared (Fig. 1) and the definitive glenoid allograft was impacted in place (Fig. 2). The glenoid allograft was additionally stabilized with 2 anchors (Super Quick anchors; DePuy Mitek, Warsaw, IN, USA) in 1 patient. A stemmed humeral HA (Anatomical; Zimmer, Inc., Warsaw, IN, USA) was then implanted in all patients, with cementation in three and without cementation in two patients. The shoulder was reduced, and the lesser tuberosity osteotomy was anatomically repaired using transosseous sutures tied over a cortical bone plate [22].

### Postoperative protocol

All patients wore a sling for 6 weeks postoperatively. For the first 6 weeks the physiotherapy protocol consisted of passive and active-assisted mobilization, with no external rotation greater than zero degrees and no internal rotation behind the back. Patients were regularly monitored at 6 weeks, 3 months, 6 months, and at 1 year after surgery or more frequently according to their clinical evolution.

### Patient evaluation

All patients had complete, standardized, physical examination and scoring Subjective Shoulder Value (SSV) [23] and relative (age-adjusted) Constant-Murley (CSr) [24, 25]) as well as standardized imaging preoperatively and at final follow-up (FU) or before revision surgery.

Pre- and postoperative radiographs and computed tomographic (CT) scans were evaluated for glenoid type according to Walch et al. [26] by 2 observers in consensus. Glenoid erosion was additionally measured on axial CT scans. For this purpose, the distance from the base of the coracoid to the glenoid articular surface was measured in the preoperative CT scans and compared with the same measurement on CT scans at latest FU or before revision.

## Results

Clinically the overall results were excellent in one, good in one and poor in three cases. Mean preoperative SSV was 34% (range: 20-50%) and preoperative CSr was 43 points (range: 29-64 points). Three patients with a poor rating had to be revised within the first 3 years. Their mean SSV and CSr before revision were 38% (range: 15-80%) and 36 points (range: 7-59 points) respectively. One patient - initially rated as good - had to be revised 9 years after the primary procedure. Nine years after the index procedure the SSV was 60% and CSr was 65 points. One patient has an ongoing satisfactory outcome without revision. Ten years after the index procedure the SSV is 83% and CSr is 91 points. Mean FU was 7 years (range: 2-10 years) and mean time to revision was 4 years (range: 1-9 years).

### Patients with an unsatisfactory course

1. The first patient, a 52-year-old woman, presented with persistent chronic pain and loss of function after sustaining a proximal humerus fracture treated with open reduction internal fixation 12 years before the index surgery. There was no major deformity of the proximal humerus but partial humeral collapse with secondary OA. According to the glenoid bone loss classification system of Walch et al. [26], the patient had a type A2 glenoid. After HA with osteochondral glenoid allograft initial pain relief and improved function were noted, but the patient worsened rapidly thereafter. The erosion of the glenoid allograft bone was 0.5 cm at latest FU 24 months postoperatively. Infection was ruled out and a revision to TSA was performed 2.5 years after the index procedure but did not alleviate symptoms. A revision to an RTSA was performed 9 months after the TSA and the patient was satisfied because of pain relief at latest FU with an SSV 35% of and CSr of 43 points 5 years postoperatively.
2. The second patient was a 37-year-old man who had had two stabilization procedures, including open and arthroscopic Bankart repair and subsequently developed OA (type B1 glenoid). Nine months after HA and glenoid allograft procedure the patient remained unacceptably painful with an SSV of 15% and a CSr of 7 points. CT showed a median erosion of the allograft of 0.3 cm. Consecutively, conversion to an RTSA was performed 9 months after the initial procedure. At last FU, 1 year after revision surgery, the pain had clinically resolved. This patient was lost to follow-up thereafter.
3. The third patient was a 51-year-old man who presented with primary OA (type B2 glenoid) with pain and loss of function. After HA with osteochondral glenoid allograft the pain persisted with an SSV of 20% and a CSr of 46 points. The median abrasion of the glenoid bone was 0.2 cm at last FU. Consecutively revision to a TSA was performed 1 year after the index procedure. Two years after glenoid conversion radiographic loosening was diagnosed. Conversion to an RTSA was offered but refused and the patient was lost to follow-up.

### Patients with a satisfactory course

**Fig. 3:**
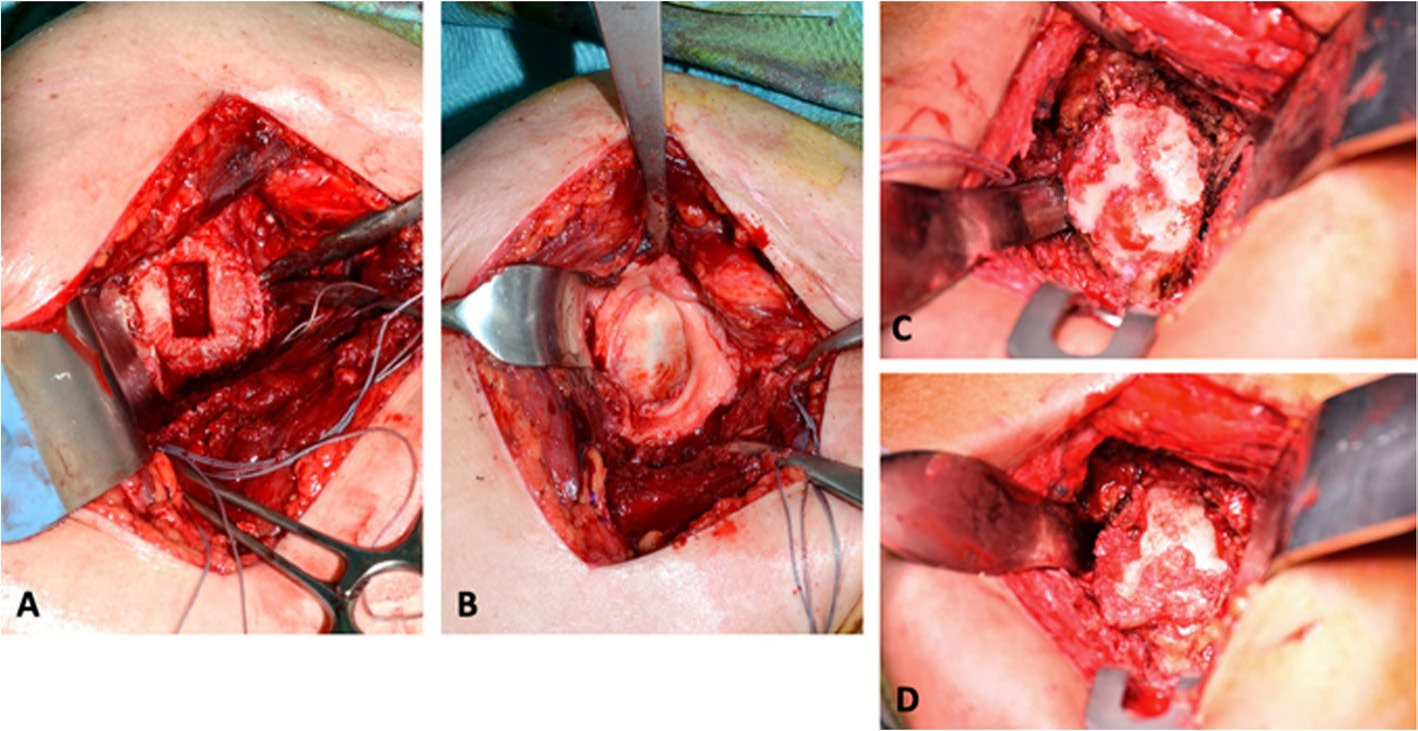
Intraoperative imaging of the patient with an initially satisfactory course but revision 9 years after index procedure. Glenoid preparation at index procedure (**A**), glenoid allograft after implantation (**B**), eroded glenoid allograft at revision surgery (**C** + **D**)

1. The first patient was a 36-year-old patient with a large glenoid defect after a direct trauma (with possible posterior (sub-)luxation) who rapidly developed OA (type B2 glenoid). Nine months after HA with glenoid allograft the pain persisted during overhead and internal rotation movements as well as at night, but the patient recovered fully and presented for 10-year FU with a SSV of 83% and a CSr of 91 points (Figs. 1 and 2).
2. The second patient was a 57-year-old woman who presented with primary OA (type A1 glenoid) with pain and loss of function. One year after HA with an osteochondral glenoid allograft she presented with persistent anterior shoulder pain especially with internal rotation but had an already improved SSV to 80% and a CSr of 59 points. Between 3 and 8 years postoperatively she had an uncomplicated and satisfactory course. Nine years after the index procedure conversion to TSA with a cemented polyethylene glenoid was performed due to a gradual increase in pain and loss of ROM. Intraoperatively bone stock was somewhat compromised, but sufficient for an anatomical restoration (Fig. 3). 4 months postoperatively the patient was very satisfied with the situation.

## Discussion

The most important finding is that the technically feasible glenoid allograft for resurfacing of a degenerated glenoid surface combined with anatomical HA does not work in-vivo.

We report the results of five consecutive osteochondral glenoid allograft resurfacings of the glenoid for the treatment of glenohumeral OA young patients performed in a single institution. After unsatisfactory results with 3 other types of biological resurfacings (i.e. capsular interposition, meniscal allograft interposition, and glenohumeral GraftJacket™ interposition arthroplasty) [13], we aimed at trying to resurface the glenoid using a glenoid allograft. The concept was experimentally tested, the design of the construct was optimized and feasibility of glenoid allografting was validated in-vitro [15]. After successful experimental validation, a limited pilot study for patients with OA and an intact rotator cuff was set up [21]. As OA of the young can be either primary or occur after previous trauma or instability we decided to use allograft resurfacing for primary or secondary OA provided the rotator cuff was intact and there was no major posttraumatic malposition of the proximal humerus or the glenoid. At the time of this trial, the use of HA or TSA in Type B glenoids was accepted. Today, primary replacement with RTSA is preferred by many although good mid- to long-term results have also been observed with TSA [27].

A 2- to 15-year review presents the results of soft tissue glenoid resurfacing in 34 patients (36 shoulders) with a mean age of 51 years treated between 1988 and 2003. Various biologic surfaces, including anterior capsule (7 cases), autogenous fascia lata (11 cases) and Achilles tendon allograft (18 cases) were used, reflecting the evolution of the technique over time. All scores performed improved postoperatively, and using Neer’s criteria, the outcome was excellent in 18 shoulders, satisfactory in 13, and insatisfactory in 5 shoulders. Unsatisfactory results were related to infection, early re-injury, and the use of the anterior capsule as interpositional material. Erosion of the glenoid averaged 7.2 mm but appeared to stabilize after approximately 5 years [28]. This contrasts with results of a study that published lateral meniscus allograft or human acellular dermal tissue matrix in 42 patients at an intermediate-term follow-up of 2.8 years. The lateral meniscal allograft cohort had a failure rate of 45.2%, with a mean time to failure of 3.4 years. Human acellular dermal tissue matrix interposition had a failure rate of 70.0%, with a mean time to failure of 2.2 years [10].

All procedures in this study were performed by a single surgeon (C.G.) using a consistent, previously laboratory tested technique. The results of 5 out of 5 patients are known and only 1 result is clinically durably good but radiographically associated with advanced glenoid erosion, a combined clinical and radiographic result well known from HA without any resurfacing [1]. A statistical analysis is not feasible as early failure with severe pain and poor function requiring revision within 3 years, one case requiring revision at 9 years and one clinically good result with an unsatisfactory radiographic course are considered prohibitive for further use of this technique. Unfortunately, no difference in clinical or radiographic parameters could be identified which differentiated the two results which were satisfactory for at least 8 years and the three poor results.

As the main limitations, the range of indications can be criticized but this corresponds to the type of OA that is seen in rather young individuals who have an intact rotator cuff. Secondly, we used commercially available, size and side matched deep-frozen allografts which were thawed in Ringer’s solution at the beginning of the surgery, and we cannot exclude that some specific preparation of the graft material could have yielded better results, but the grafts used correspond to the standard used in other joints or indications. In addition, it may be criticized that there was no control group for this pilot study. The study, however, was designed to determine the safety and suitability of the procedure for a further RCT.

The early mid- and long-term results of HA and TSA for OA in relatively young patients are not yet where patients and surgeons would like them to be, but they are well established [1]. With a follow-up of 3 to 13 years, the overall implant survivorship is between 60 and 90%. Glenoid loosening is the most common reason for revision (52%). A reliable improvement of the subjective as well as objective outcome can be expected, which possibly decreases between mid- and long-term follow-up [29, 30]. Glenoid resurfacing using allograft is a theoretically attractive solution which can technically reproducibly be executed in-vitro. In-vivo, however, it led to early revision in three out of 5 cases due to severe pain, and to a result no better than the appropriately documented results of HA and TSA in the two best cases so that we have abandoned this concept.

## Conclusions

We conclude that the technically feasible glenoid allograft for resurfacing of a degenerated glenoid surface combined with anatomical HA does not work in-vivo. The results in young individuals are inferior to those of HA and TSA.

Glenoid allograft resurfacing with prosthetic humeral head replacement is not a viable alternative for the treatment of advanced OA in the young individual due to its high risk of failure.
